# The Effect of Menopause on Antipsychotic Response

**DOI:** 10.3390/brainsci12101342

**Published:** 2022-10-04

**Authors:** Alexandre González-Rodríguez, José A. Monreal, Mary V. Seeman

**Affiliations:** 1 Department of Mental Health, Mutua Terrassa University Hospital, Fundació Docència i Recerca Mutua Terrassa, University of Barcelona (UB), Centro de Investigación Biomédica en Red de Salud Mental (CIBERSAM), 08221 Terrassa, Spain; 2 Institut de Neurociències, Universitat Autònoma de Barcelona, 08221 Terrassa, Spain; 3 Department of Psychiatry, University of Toronto, Toronto, ON M5P 3L6, Canada

**Keywords:** antipsychotics, drug metabolism, menopause, schizophrenia

## Abstract

*Background*: It has been hypothesized that, whenever estrogen levels decline, psychosis symptoms in women increase. At menopause, this can happen in two main ways: (a) the loss of estrogen (mainly estradiol) can directly affect central neurotransmission, leading to increase in schizophrenia-related symptoms, and (b) the loss of estrogen can decrease the synthesis of enzymes that metabolize antipsychotic drugs, thus weakening their efficacy. *Aims* *and Methods*: The aim of this narrative review was to investigate the second possibility by searching PubMed and ClinicalTrials.gov for studies over the last two decades that investigated the metabolism of antipsychotics and their efficacy before and after menopause in women or that studied systemic and local estrogen level effects on the pharmacokinetics and pharmacodynamics of individual antipsychotic drugs. *Results*: The evidence suggests that symptom level in women with schizophrenia rises after menopause for many reasons beyond hormones but, importantly, there is an estrogen-dependent loss of efficacy related to antipsychotic treatment. *Conclusion*: Effective clinical intervention is challenging; nevertheless, several promising routes forward are suggested.

## 1. Introduction

### 1.1. Differences in Short and Long Outcomes in Women with Schizophrenia

The literature on short term treatment outcomes (under 10 years) that compare men and women with schizophrenia consistently point to female advantage. Compared to men, women are shown to have fewer and shorter hospitalizations plus a lower rate of suicide, homelessness, substance abuse, and forensic involvement [1].

Women’s family and social relationships are reported as superior to men’s [2,3], and women are generally found to exhibit a more robust response to treatment [4]. Importantly, however, long term outcomes (over 10 years) are similar in men and women [5]. Equally important are data that show women with schizophrenia, but not men, experiencing increases in symptom severity at midlife (over age 40) [6]. Increases in cognitive symptoms and functional decline over time are often attributed to age, but positive, negative, and affective symptoms also worsen. Two main potential explanations for increased symptoms in women post menopause are (a) estrogen loss directly affects neurotransmitters that mediate those symptoms and (b) estrogen loss moderates the efficiency of enzymes that metabolize antipsychotic drugs such that less of the drug arrives at target sites in the brain [7].

### 1.2. Why Estrogen Is Important

Three estrogens (estrone, 17β-estradiol (E2), and estriol) regulate many central nervous system functions [8] and operate through three estrogen receptor subtypes, Erα, Erβ and G-protein-coupled estrogen receptor 1 (GPER). The three ER subtypes are genetically distinct and mediate different estrogenic actions. ERα and Erβ, initiate both genomic and non-genomic traditional sexual and reproductive functions while GPER mediates neuroprotection and cognition [8].

Estrogens are synthesized from the parent molecule, cholesterol, with estradiol (E2) being the most potent of the three [9,10]. E2, mainly produced in ovarian oocytes, gradually decreases over the course of perimenopause [11]. Estrone (E1) is mainly synthesized in the adrenal glands and adipose tissue and continues to be available after menopause. Estriol (E3) is mainly produced during pregnancy. Estrogens originate in many body sites, including the brain.

At menopause (the cessation, in women, of menses due to the death of oocytes), many women experience cognitive problems because the decline in E2 is associated with a reduction in the volumes of both the hippocampus and the parietal cortex [11]. There are other effects of estrogen decline that can lead to a variety of symptoms at this time because E2, the estrogen that is lost at menopause, serves many functions. It insulates against the effects of injury, inflammation, ischemia and apoptosis; it activates signal transduction pathways and modulates intracellular homeostasis. It also promotes neuronal growth and activates other neuroreparative processes such as decreasing blood–brain barrier permeability, reducing oxidative stress, neuroinflammation and excitotoxicity, and promoting synaptic plasticity, axonal growth, neurogenesis, and remyelination [12].

### 1.3. The Perimenopause

There are two stages to the years leading up to menopause—the early menopausal transition when menstrual cycles remain mostly regular, and the late transition, where amenorrhea becomes more prolonged and lasts for at least 60 days until it stops altogether [13]. Perimenopause usually lasts from 7 to 14 years during which, in the early phase, estrogen levels may paradoxically increase [14]. This is because, in order to stimulate estrogen production, follicle-stimulating hormone (FSH) secretion rises in response to decreasing levels of oocyte estrogen secretion [15]. Most women are able to accommodate to estrogen level fluctuations during this period because of prior exposure to menstrual period fluxes, the 9-fold gradual rise of estrogen over the course of pregnancy [16] and the precipitous drop postpartum. This sharp, abrupt fall precipitates postpartum psychosis in approximately one woman in a thousand [17]. The drop at menopause is more gradual but no less significant and, in a substantial number of women with schizophrenia, is associated with an increase in the severity of psychotic symptoms [18,19,20].

### 1.4. Estrogen, Dopamine, and Schizophrenia

The menopausal risk of increased psychosis severity may be tied to the link between estradiol and brain dopamine [21,22], the neurotransmitter most closely implicated in the pathogenesis of schizophrenia [23]. Relatively little is known about sex differences in dopamine function but D2/D3 dopamine receptors, the neuronal membrane receptors to which most antipsychotic medications preferentially bind [24] do show some differences between men and women. The results of human PET studies show that women have more D2 receptors than men in the frontal and temporal cortex and in the thalamus. In the striatum, D2 receptor density declines with age faster in men than in women. Animal studies also point to similar sex differences [25].

One research group studied sex differences in D2 receptor occupancy after administration of the antipsychotic drug, olanzapine, and demonstrated that women are able to achieve the same D2 receptor occupancy as men while taking a lesser daily oral dose [26]. Current thinking is that the dopamine transporter that returns dopamine to its presynaptic cell after secretion into the synaptic gap is key to the hyperdopaminergic state responsible for psychotic symptoms, and estradiol activity has been found at the transporter site [27]. It is thought that the lower the estrogen level in relevant parts of the brain, the more severe the psychosis [28]. The amount of antipsychotic medication that reaches the brain is determined by its level in the plasma, and that, in turn, is chiefly influenced by liver and gut enzymes that break down antipsychotic drugs. Relevant to our review is the fact that estradiol levels can speed or slow some of the enzymes that metabolize specific antipsychotic drugs [29,30]. This effect is most evident when the drugs are administered orally but still affects intramuscularly or intravenously administered antipsychotics.

Further suggestive evidence of the potential link between estrogen and dopamine transmission is the well-established fact that women first express the symptoms of schizophrenia at a later age than men [31,32]. Important also is the high incidence rate of schizophrenia in women with Turner’s syndrome [33,34]. Turner’s syndrome (one X chromosome wholly or partially missing) is a chromosomal abnormality characterized by reduced serum estrogen levels. Further evidence is that both male and female schizophrenia patients show reduced levels of plasma estrogen when compared to age and sex peers [35]. Another confirmatory finding is that early puberty in girls (but not in boys) is associated with relatively later onset of symptoms in young people who go on to develop schizophrenia [36,37,38]. It is also known that symptoms fluctuate over the menstrual cycle in women with schizophrenia, increasing in severity when estrogen levels are low [29,39]. At postpartum, when estrogen levels precipitously drop, women with schizophrenia often relapse [40]. An interesting epidemiological finding is that the relative male/female incidence of schizophrenia reverses after age 40 [41]. There is also evidence that estrogen-reducing drugs trigger acute psychosis [42]. Perhaps most convincingly, adding estrogen or selective estrogen receptor modulators (SERMS) to an antipsychotic regimen improves treatment efficacy [43].

### 1.5. Estrogen and Antipsychotic Efficacy

Duffy and Epperson concluded that estrogen levels influenced both the efficacy and the side effects of antipsychotic drugs after reviewing 12 human brain imaging studies that investigated sex differences in antipsychotic response [44]. None of the 12, however, were looking for a menopausal effect. It has been clinically noted that, after menopause, the dose of the antipsychotic medication often needs to be increased in women to maintain symptom stability. This is either because symptoms have become more severe or because the drugs function less well. The time duration since menopause has been shown to correlate negatively with antipsychotic response in postmenopausal women. The results of a 12-week prospective study of 64 postmenopausal women diagnosed with schizophrenia [7] used the duration of reproductive years (menarche to menopause) as an indirect measure of cumulative estrogen exposure. The antipsychotic response was defined as a reduction of at least 30% on the Positive and Negative Syndrome Scale. Antipsychotic adherence was assessed by plasma level monitoring at 4 weeks. Forty-two participants (66%) were found to be antipsychotic responders. Time since menopause was significantly and negatively associated with antipsychotic response, explaining almost 42% of the variance. This finding could be a consequence of a direct effect of estrogen loss on dopamine transmission and, thus, on psychotic symptoms, or, alternatively, a direct effect of estrogen loss on drug metabolism and, hence, on plasma levels of antipsychotics entering the brain.

### 1.6. Estrogen after Menopause

Estradiol levels continue to fluctuate to some degree after menopause. Estrone continues to be produced in the adrenal glands (until adrenopause) and in adipose tissue, and estrogens are synthesized in bone, vascular endothelium, aortic smooth muscle cells, and in numerous sites in the brain [45,46]. In women after menopause, estrone levels increase from premenopausal levels. Estradiol (E2) levels are the ones that drop [9,10].

Estradiol is synthesized in both neurons and glial cells and is classified as a neurosteroid. Neurosteroids directly modulate plasma membrane ion channels and regulate intracellular signaling and they exert powerful effects on the brain before and after menopause [47]. Of relevance to the effect of estrogen in schizophrenia is the distribution of estrogen receptors in the brain. They are most abundant in the subcortical structures that are involved in schizophrenia (the hippocampus, amygdala, thalamus, and nucleus accumbens) and within neurotransmitter pathways (dopaminergic, serotonergic, and glutamatergic), all of which are implicated in the pathogenesis of schizophrenia symptoms [48].

### 1.7. Symptom Exacerbation after Menopause Due to Stress

We have not yet mentioned a potential explanation for worsening symptoms and the need for increased antipsychotic dosing in women with schizophrenia after menopause that is independent of estrogen loss. Menopause itself is a stressful time for women. The physiological symptoms of menopause (vasomotor, sleep, sexual, and cognitive) are distressing. There are further psychological and social pressures at this time (children leaving home, parents dying, marital, employment and economic constraints). The impact of fertility loss, the need to adapt to aging, and the onset of a variety of new medical conditions further augment the stress experienced by menopausal women [18]. The effect of stress is not altogether independent of estrogenic influence, however, since the hypothalamic-pituitary-adrenal stress axis (HPA-axis) responds, to some degree, to estrogen. This has been well shown in animal studies, but the results in human studies are more controversial. Men consistently show greater HPA reactivity than women when evaluated for achievement. Some studies have found greater reactivity in women when the stressor is social performance. Sex difference (and, by implication, estradiol level) appears to depend on the nature of the stress [49]. Important to note is that both antipsychotics and stress increase prolactin levels, which, in turn, decreases estrogen levels [5].

### 1.8. Aims

The specific aim of this review paper is to examine to what extent the loss of antipsychotic efficacy in women with schizophrenia after menopause is explained by estrogen’s effect on metabolizing enzymes. We review the literature

(a) On the contrast in antipsychotic effectiveness pre- and post-menopause in women with schizophrenia;
(b) On systemic and local estrogen level effects on the pharmacokinetics and pharmacodynamics of individual antipsychotic drugs.

## 2. Methods

We carried out a narrative review based on electronic searches on PubMed of papers investigating metabolism of antipsychotics and antipsychotic efficacy at and after menopause.

We used the following search terms: antipsychotics AND (pharmacokinetics OR pharmacodynamics) AND estrogen; (menopause OR postmenopausal OR estrogen) AND schizophrenia AND antipsychotics. We targeted studies published over the last two decades; however, some older papers were also considered if they were frequently cited and relevant to our aims. Additionally, ClinicalTrials.gov was searched for relevant papers not found in the PubMed search. Reference lists of the initially selected papers were screened for frequently cited older articles.

Inclusion criteria: (1) language restricted to English, French, Spanish and German, (2) publication in peer-reviewed journals, (3) focus on effectiveness of antipsychotics in postmenopausal women or (4) focus on sex differences in pharmacodynamics and pharmacokinetics of antipsychotics. Exclusion criteria: (1) case reports or case series, (2) patient samples receiving antipsychotics but diagnosed with affective or organic medical conditions.

The screening of several hundred abstracts (N = 552) and the selection process for full length paper screening and ultimate inclusion were performed by all authors. Disagreements about inclusion were resolved by group discussion.

**Figure 1:**
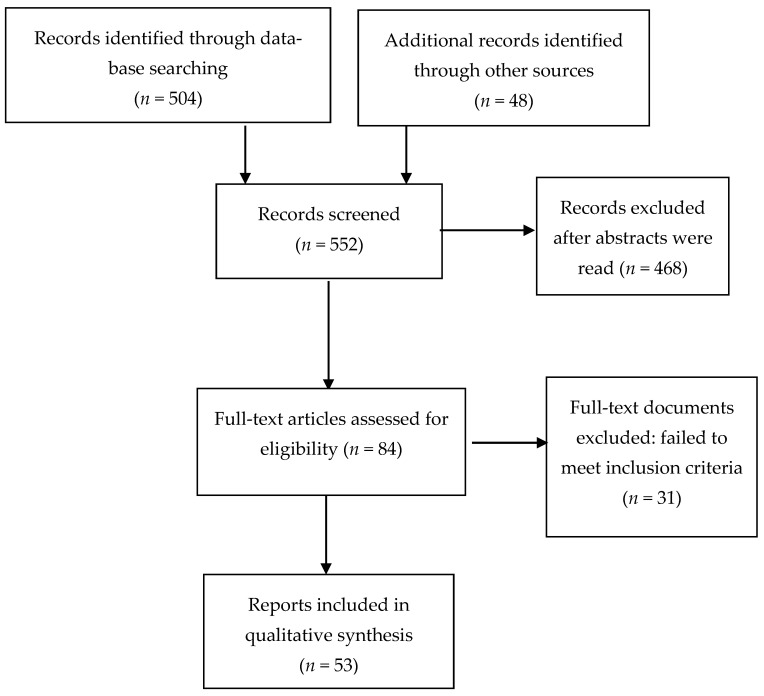
Flow diagram of included studies.

Figure 1 shows the flow chart of papers screened and those excluded. In the end, a total of 53 studies were included in the review. 

## 3. Pharmacokinetics of Antipsychotics

### 3.1. Absorption of Antipsychotics

Absorption, but also distribution, metabolism and elimination are affected by women’s reproductive phases: puberty, menstrual stages, pregnancy, lactation, and menopause. The oral route for absorption is the most common one for antipsychotic drugs, although long-acting depot antipsychotics are becoming popular, and they are administered intramuscularly [50].

Numerous sex differences in oral drug absorption (effect on gastric acid, gastric emptying time, intestinal contractility and transit time) have been identified and are usually attributed to hormonal differences between men and women that wane at older ages [51]. Recent research suggests, however, that some sex differences in drug bioavailability may persist throughout life. Castberg et al. [52] tested the absorption vs. elimination of several antipsychotic drugs—clozapine olanzapine, risperidone and quetiapine. They found that host age predicted the concentrations of all four drugs. At patient age 80, concentrations of drugs adjusted by dose were almost twice as high as at age 40, suggesting increasingly impaired rates of elimination. Concentration increase by age was highest for clozapine and lowest for olanzapine, suggesting differences between the two drugs in 1st and 2nd pass metabolism. Women showed higher concentrations than men (20–30% higher) at both pre- and post-menopausal ages, implying that peripheral estrogen levels are not the only factors that account for sex differences in bioavailability.

With respect to intramuscular injections, they are reportedly more successfully administered in men than in women. This depends on the depth of fat covering the muscle at the site of the injection and the length of the needle used [53]. This means that women may not benefit as much from injections as men do.

### 3.2. Antipsychotic Drug Metabolism

While absorption, distribution, protein binding, and elimination of antipsychotics all differ by sex and age [29], the key to understanding antipsychotic response in women at menopause is the effect of estrogen plasma levels on the cytochrome P450 (CYP) enzymes that metabolize antipsychotics, mainly in the liver and gut [54,55]. It is not always possible, however, to definitively identify which CYP enzyme is involved in the metabolism of a particular drug because, for the most part, in vivo data come only from individuals case reports [54].

For most 2nd generation antipsychotics, the principal metabolizing enzymes are currently considered to be: aripiprazole CYP 2D6 and CYP 3A4; asenapine CYP 1A2 and CYP 2D6; clozapine CYP 1A2; iloperidone CYP 2D6 and CYP 3A4; lurasidone CYP 3A4; olanzapine CYP 1A2; quetiapine CYP 3A4; risperidone CYP 2D6; ziprasidone CYP 3A4 [55]. Specific CYP activity can be inhibited or induced by concomitant drugs or by levels of endogenous or exogenous hormones [56,57]. Estrogen levels change the activity of the following liver and intestinal metabolic enzymes: CYP1A2 (asenapine, clozapine, olanzapine), CYP2C9, CYP2C19, and CYP3A4 (aripiprazole, iloperidone, quetiapine, ziprasidone) [58].

According to the above, doses of aripiprazole, iloperidone, quetiapine, and ziprasidone need to be lowered at and after menopause to prevent adverse effects while doses of asenapine, clozapine and olanzapine need to increase to maintain effectiveness. Cigarette smoking induces CYP 1A2, so menopausal women who smoke and are taking drugs metabolized by CYP 1A2 need significantly raised doses but, should they cease smoking, the dose needs to be adjusted downwards [55,58,59]. Those taking drugs metabolized by CYP2C9, CYP2C19, and CYP3A4 may suffer increased side effects after menopause.

Sex hormones also influence phase II metabolism (glucuronidation, sulfation, acetylation, methylation, glutathione conjugation), although the relevant data with respect to antipsychotics are sparse [60]. It is to be noted, however, that female contraceptives affect phase I and phase II metabolism in opposite directions [61].

A relatively neglected contributor to drug pharmacokinetics is emerging from studies of differences in the composition of male and female microbiomes [62,63,64]. Different gut bacteria produce metabolic enzymes that are not necessarily identical. The most dominant biotransformations ascribed to bacterial enzymes in the gut involve reductive metabolism (the antipsychotic, risperidone is an example) and hydrolytic reactions and, to a lesser extent, decarboxylations, dehydroxylations, dealkylations, dehalogenations, and deaminations [65]. Hormones such as estrogen regulate metabolizing enzymes in gut bacteria as well as in human liver [66]. This has raised the possibility that menopause alters male/female composition of gut bacteria and the actions of their metabolizing enzymes [67]. Indeed, several observational studies have confirmed that the gut microbiome is influenced by the hormonal environment [68]. These investigations, however, are still at an early stage.

### 3.3. Antipsychotic Drug Distribution—Effect of Adipose Tissue Changes Post Menopause

Because of the action of female hormones on fat cells, women’s bodies, in general, have a greater amount of fat mass than men, the difference decreasing after menopause [51]. This affects the distribution of antipsychotics, which are lipophilic drugs. Changes in drug distribution affect both the effectiveness and safety of drugs [69].

Once ovaries no longer produce estradiol (i.e., after menopause), estrogens continue to be produced from cholesterol in many tissues, but especially in adipose tissue. Lipid stores in women change from their principal premenopausal subcutaneous location (mainly in the thighs) to the abdomen, which then makes distribution similar to that of men [69,70,71], a pattern that has been associated with heightened risk for adverse effects such as diabetes and cardiovascular disturbance [72,73]. Thus, not only the efficacy but also the safety of antipsychotic drugs changes after menopause in women, issues that need testing in clinical trials [74].

### 3.4. Elimination of Antipsychotics in Men and Women

The sexually dimorphic GI microbiome may affect drug elimination differently in women and men [75]. Renal elimination depends on tubular secretion, reabsorption, and glomerular filtration rate, which are reportedly lower in women than in men [76]. It also depends on body weight [76]. In one study of human kidney, 23 genes coding for drug transporters showed differential male/female mRNA expression. Twenty-one of these genes were expressed at higher levels in men, whereas two were expressed at higher levels in women [77].

### 3.5. Genes and Antipsychotic Response

Importantly, individual gene polymorphisms determine antipsychotic response because they bear on all aspects of pharmacokinetics, especially metabolism [78,79].

Alkelai et al. used whole-genome sequencing to analyze variants of CYP2D6, responsible to a large degree for the metabolism of aripiprazole, asenapine, iloperidone, and risperidone [79]. They identified 57 different genotypes that predicted five metabolic phenotypes (poor metabolizers to ultra-rapid metabolizers). Genetic variants have also been reported in enzymes responsible for the metabolism of clozapine [80]. A current summary of drug-gene variations is available [81]. We know from the results of Yang et al., who recorded gene expression of 374 drug-metabolizing enzymes and transporters in male and female liver samples, that at least 77 such genes differ in the two sexes [82]. Sex differences in pharmacokinetics that persist post menopause [83,84] could result from a combination of genetic factors, e.g., genetic sex difference in drug metabolism independent of estrogen, genetic disparity between men and women in the volume of drug distribution, and genetic sex differences in glomerular filtration rates [85]. Furthermore, there are many variants of the dopamine receptor D2 (DRD2) gene [86] and the 5-HTR2A gene [87] that may theoretically affect antipsychotic response. In all patients receiving antipsychotic medications, genetic variations in both metabolic genes and neurotransmitter genes play a role in response.

Individual sex differences that are not attributable to hormones or genes can also result from disease states, diet and environmental exposures, all of which can change after menopause.

### 3.6. Pharmacodynamics of Antipsychotics

Pharmacodynamics refers to how a drug works once it reaches the site of its intended effect (membrane receptors, ion channels, relevant enzymes, and signaling pathway), but also how it impacts other, non-targeted body sites where it can sometimes exert unwanted effects [88]. For instance, a significant pharmacodynamic sex difference is the increased prevalence of QT interval prolongation in women and men (but worse in women) that is induced by many antipsychotics. This leads to an increased incidence of tachycardias, arrythmias and syncope in women. An early study investigated 32 cases of antipsychotic-induced Torsades de Pointes and found that women constituted 70% of all cases [89].

A drug’s efficacy with respect to receptor binding depends on its molecular configuration; in the case of antipsychotics, this would refer mainly to D2/D3 receptors [88]. Psychodynamic effectiveness is also affected by host age, sex, frailty status and associated hormone levels, immune/inflammatory characteristics, microbiome composition, and genetic factors.

Estrogen levels in the brain determine, in part, the integrity of dopamine pathways and influence functional connectivity, neurotransmission, and brain structure [29,90,91,92,93]. The threshold percentage of D2/D3 receptors that antipsychotics need to occupy for effectiveness, and the threshold that results in extrapyramidal adverse effects, is modulated by sex. Kaasinen et al. [94], using positron emission tomography (PET) and a high-affinity radioligand to measure extrastriatal D2-like receptors, evaluated D2 binding potential in the frontal cortex, temporal cortex, and thalamus of 12 healthy men and 12 healthy women. Women showed higher D2 binding in all three regions, but the difference relative to men was only statistically significant for the frontal cortex. A more recent register-based study [95] investigated how age and sex influenced striatal D2 receptor availability using [11C] raclopride. The pooled data from 5 different PET scanners yielded information on 120 healthy males and 36 healthy females, aged 19 to 71. Binding consistently declined with increasing age in both sexes, most convincingly in men and women between the ages of 20 and 60, the range for which there were most data. The decline occurred in both sexes but, on average, female binding stayed between 6–8% higher than male binding irrespective of age. Lateralization was more evident in males than in females. The investigators conducted a replication study of 135 participants that showed the same age and sex effects as the first study. The confirmation is important because their findings were not altogether in harmony with earlier results reporting sex-dependent decline in dopamine function, with males showing steeper reduction in receptors [96,97]. Using radioactive olanzapine as their PET ligand, Eugene and Masiak [26] had found, like Malén et al. [95], that, over various age ranges, women needed a smaller oral dose than men to achieve an occupancy of 70% D2 binding, 60% being the usual minimum required for antipsychotic efficacy. The amount of DA transporter (DAT) has been found to be higher in postmenopausal women compared to similarly aged men (age range 50–86, mean 70 years) [98]. Others had previously also found sex differences in DATs [99,100]. In addition, it has been shown that women show significantly higher presynaptic dopamine synthesis than men in the striatum, more in the caudate than in the putamen [101].

Importantly, D2 receptor availability has been reported to vary with the level of plasma sex steroids [102,103]. Sex-specific hormones as well as genes evidently play a role in dopaminergic function [104] and this implies that menopause, when estrogen levels sharply decline, changes women’s response to antipsychotics. Menopause may also affect the severity of adverse effects. In a cross-sectional study, Iversen et al. [105] found that over 75% of individuals taking antipsychotics reported adverse effects and that twice as many women as men described these as severe. Ages and hormonal status were not specified in this study. The adverse effects that are most likely to be impacted by sex hormones and, therefore, by menopause are: extrapyramidal effects (including tardive dyskinesia), agranulocytosis, cardiac arrythmia, metabolic effects, and hyperprolactinemia. According to hospital statistics, these adverse effects of antipsychotics are, in general, more prevalent in women than in men [106], but how much of this is due to menopause has never been investigated.

Finally, epigenetic modifications can play a role in male/female pharmacodynamic differences because DNA methylation and histone acetylation differ between the two sexes [107,108].

### 3.7. Changes in Postmenopausal Clinical Response to Antipsychotics

Because estrogen, via its effect on dopamine pathways, is thought to enhance the efficacy of antipsychotics, psychotic symptoms increase in severity after menopause and, correspondingly, women become less responsive to antipsychotics than they were during reproductive life. This means that, frequently, doses need to be raised and side effects, therefore, become more likely.

A small 3-year survey from the 1980s explored the interaction between sex, age and dose in patients receiving antipsychotics [109]. Men at younger ages required higher doses than women matched for age. By the time of menopause, however, women needed higher doses of antipsychotics than men of comparable age, and higher doses than younger women. Many years later, González-Rodríguez and collaborators [7] carried out the prospective observational study referred to earlier in this review. Participants were 64 women with schizophrenia or closely related disorders. All were postmenopausal and all required a change of antipsychotic because of failure to respond. For the assessment of psychotic symptoms and of function, the Positive and Negative Syndrome Scale (PANSS), the Clinical Global Impression Scale (CGI), and the Personal Social Performance Scale (PSP) were used. Cumulative estrogen exposure, calculated as the difference between age at menarche and age at menopause, was not predictive of overall antipsychotic response. Time since menopause, however, was negatively correlated with antipsychotic response, which suggested that the longer the time since menopause, the poorer the response to antipsychotics. This implies that, besides estrogen loss, age and increasing medical challenges may play important roles in antipsychotic response in this population.

The same research group investigated the association among gonadal hormones, follicle-stimulating hormone (FSH), luteinizing hormone (LH) and FSH/LH ratio and clinical improvement in postmenopausal women with schizophrenia [110]. Because increasing levels of FSH are a marker of poor ovarian reserve in women, the FSH/LH ratio has been used in reproductive medicine to predict response to controlled ovarian stimulation and outcomes of in vitro fertilization procedures. The study authors hypothesized that FSH/LR ratio would correlate positively with antipsychotic response in postmenopausal women. Thirty-seven acutely ill postmenopausal women with schizophrenia received a newly initiated antipsychotic as part of a 12-week prospective observational study. PANSS, CGI and PSP were administered at baseline and again 12 weeks later. After correcting for potential confounders and multiple testing, there was no significant association in serum levels of estradiol, progesterone, testosterone, FSH, LH or FSH/LH ratio with clinical improvement. Prior to Bonferroni correction, however, the FSH/LH ratio did correlate positively with improvement, so that this remains a promising area of investigation.

Table 1 summarizes changes in clinical outcomes in women with schizophrenia after menopause.

In summary, the literature suggests that clinical status worsens and antipsychotic response decreases after menopause in women. Many postmenopausal women with schizophrenia require higher doses of antipsychotics than their premenopausal peers [109,110,111]. The progressive decline of response to antipsychotics is partly explained by estrogen decline, but the evidence suggests that other factors are implicated as well.

Table 2 summarizes the main factors associated with variations of clinical response in postmenopausal women with schizophrenia.

## 4. Discussion and Future Directions

The estrogen protection hypothesis postulates a neuroprotective role for estrogens in the brain that shields it, to some extent, from the development of psychotic symptoms. The decline of estradiol at the time of menopause correlates with an increase (relative to men) in the incidence of schizophrenia in women after menopause. Symptoms in those with a pre-existing schizophrenia diagnosis intensify at this time. Although several ways in which estradiol loss at menopause can increase symptom severity have been identified, this review has focused on the theoretical effects of estrogen loss on the pharmacokinetics and pharmacodynamics of antipsychotics. This is a complex area which involves estrogen effects on 1st and 2nd pass liver enzymes, and on neurotransmitter receptors in the brain. There is reason to believe that some antipsychotics are more affected by estrogen loss than others. Furthermore, from a pharmacodynamic point of view, brain estradiol levels influence the integrity of dopamine pathways, neurotransmission, brain structure and functions.

Raloxifene, a selective estrogen receptor modulator (SERM), has been found to exert neuroprotective effects in the central nervous system and has proven to be a good adjunctive drug for psychotic symptoms in the menopausal period. Most recently, a study carried out by Huerta-Ramos and collaborators (2020) investigated the efficacy of raloxifene as a treatment of cognitive symptoms when added to antipsychotics in postmenopausal schizophrenia [112]. The addition of raloxifene 60 mg/daily did not show any effect on cognitive function but improved negative and general psychopathological symptoms [113]. Recent meta-analyses have reported that raloxifene appears to be efficacious and safe for women at the time of menopause, particularly for those whose symptoms are not severe [114,115].

Clinical recommendations are:

1. When the efficacy of previously effective antipsychotic doses wanes at menopause, raising the dose is not the treatment of choice [89,93] because it increases the risk of weight gain, cardiovascular and cerebrovascular events.
2. Changing to an antipsychotic that is less affected by estrogen loss may work better, [56,57,59] because estrogen levels change the activity of CYP1A2 and CYP3A4, which principally affects clozapine and olanzapine. The work of Hoekstra et al. [116], suggests that amisulpride and aripiprazole work well post menopause.
3. Changing to a depot or skin patch antipsychotic that obviates 1st pass metabolism will improve levels of drugs affected by of CYP1A2 and CYP3A4 enzymes.
4. Adding hormone replacement or a selective estrogen receptor modulator such as raloxifene or newer SERMS or including phytoestrogens (bioidenticals) in the diet may prove effective [112,113,114,115].
5. Weight maintenance may be important because extra adipose tissue yields more estrogen [69,71] although this will be estrone rather than estradiol.
6. High prolactin levels, whether induced by antipsychotics, other drugs, or by stress, reduce estrogen levels. Prolactin-sparing antipsychotics are recommended [117].

Our findings also suggest several promising strategies for future drug trials:

(1) Comparing the effectiveness of different antipsychotics in postmenopausal women with schizophrenia
(2) Recruiting pre- and post- menopausal women in trials of antipsychotic drugs.
(3) Stratifying by hormonal status when analyzing results of antipsychotic trials [118]
(4) Comparing D2/D3 occupancy in pre- and post-menopausal women and matched age men in patients being treated for schizophrenia
(5) Comparing antipsychotic side effects in pre- and post- menopausal women and men with schizophrenia [119,120].
(6) Studying the effect on antipsychotic requirements of adrenopause in postmenopausal women and men with schizophrenia
(7) Studying the effect of sex hormones on the blood–brain barrier for antipsychotic medications [121]
(8) Studying the effect of phytoestrogens [122] in the diet and xenoestrogens [123] in the environment on symptoms and antipsychotic requirements in postmenopausal women and men with schizophrenia.

Lastly, the findings suggest a need for different antipsychotic dosing guidelines for men and women (pre- and post-menopausal).

Research recommendations: Information on pharmacogenetics and the use of ‘big data’ will inform future research. New genetic editing techniques applied to new animal models can aid in resolving questions about best treatments for postmenopausal women. Neuroimaging techniques that explore new ways of visualizing brain circuitry will help to pave the way toward precision psychiatry [124].

## 5. Conclusions

Menopause is a critical period of a woman’s life characterized by a significant decline in estrogen plasma levels. It is associated with a worsening of psychotic symptoms in women with schizophrenia. This is at least in part due to a direct effect on the brain of a decline in estrogen neuroprotection and an indirect effect of estrogen loss on antipsychotic pharmacokinetics and pharmacodynamics. Other factors, such as aging, co-morbidity, drug interactions, and the psychosocial stresses that are associated with menopause all play additional roles in the severity of women’s psychotic symptoms after menopause.

We have considered several reasons why psychotic symptoms worsen in women with schizophrenia at menopause. Two important ones are: (a) the low level of plasma estradiol that directly affects central neurotransmission, and (b) the low level of plasma estradiol that affects the synthesis of metabolizing enzymes, affecting the levels of some antipsychotics more than others.

It is important in clinical trials of women participants to investigate and aggregate data according not only to sex, but also to hormonal status. Guidelines should provide a concise and precise description of up-to-date experimental evidence, including sex-and-age-specific prescribing information that takes hormone levels into account.

## Figures and Tables

**Table 1:** Effects of menopause on clinical outcomes in women with schizophrenia.

.	Premenopausal Women	Postmenopausal Women
Antipsychotic dose	Women require lower doses of antipsychotics than men	Need higher doses of antipsychotics than premenopausal women
Clinical symptoms	Women have fewer negative and cognitive symptoms than men	Show increased psychotic symptoms Symptoms worsen with postmenopausal duration
Treatment response	Overall response is better in women than in men	Antipsychotic response worsens at menopause and continues to worsen with time

**Table 2:** Changes in clinical response in postmenopausal women with schizophrenia.

Authors and Year of Publication.	Study Design	Sample Size	Hormone Determinations	Psychopathology Assessment	Main Results
Seeman 1983 [109]	Three-year longitudinal survey (1978–1981)	N = 101 F = 43 M-58	No	Research Diagnostic Criteria (RDC)	Dose distribution identical in m and f over 3 years f/m dose ratio increased with age
González-Rodríguez et al. 2016 [7]	12-week prospective observational study	N = 64	Clinical data correlated with reproductive variables (age at menarche, age at menopause, reproductive years, time since menopause	Baseline and 12-week follow-up: Positive and Negative Syndrome Scale (PANSS), Clinical-Global Impression- Schizophrenia Scale (CGI-SCH)	Time since menopause was negatively associated with antipsychotic response. Antipsychotic response worsened with postmenopausal duration.
González- Rodríguez et al. 2017 [110]	12-week prospective observational study	N = 37	estradiol progesterone testosterone FSH, LH, FSH/LH ratio	Baseline and 12-week follow-up: (PANSS), (CGI-SCH)	Gonadal hormones and FSH/LH ratio not associated with clinical improvement at menopause

## Data Availability

The data presented in this review are available on request from the corresponding author.
